# The influence of cultural beliefs on the utilisation of rehabilitation services in a rural South African context: Therapists’ perspective

**DOI:** 10.4102/ajod.v4i1.128

**Published:** 2015-03-26

**Authors:** Liezel Wegner, Anthea Rhoda

**Affiliations:** 1 Department of Physiotherapy, University of the Western Cape, South Africa

## Introduction

South Africa has a population of 51.8 million people of which 7.5% over the age of five has a disability according to the latest census data (Statistics South Africa 2014). This statistic on the national prevalence of disability should be interpreted with caution since psychosocial and neurological disabilities are not accounted for (Statistics South Africa 2014). The most recent data on disability in South Africa is from the national census of 2011, which defined ‘disability’ as:

… a physical or mental handicap which has lasted for six months or more, or is expected to last at least six months, which prevents the person from carrying out daily activities independently, or from participating fully in educational, economic or social activities. (Statistics South Africa 2014)

In South Africa 38% of the population resides in rural areas, and 25% of the labour force is unemployed (The World Bank 2014). At the time of the national census in 2011, more than a quarter (26.3%) of all poor people in South Africa resided in KwaZulu-Natal (KZN), most living below the per capita upper-bound poverty line of R620 per month (Statistics South Africa 2014). The co-existence of poverty and disability reinforces one another (Grech 2009; Sala-i-Martin 2005). High levels of poverty together with the high incidence of disability and the large percentage of the population living in rural areas, present challenges to providing ‘health for all’ in South Africa (Department of Health [DOH] 2010; Schaay & Sanders 2008).

### Rural health

Equitable access to health care is a right of every person with a disability (United Nations [UN] 2008; Heapa, Lorenzo & Thomas 2009). A number of barriers to access in rural areas such as long distances to hospitals or clinics and poor public transport have been identified in the literature (Beatty *et al*. 2003; Harris *et al*. 2011; Maart *et al*. 2007). In South Africa the attitudes of society, and practices and ideologies, have been highlighted as important environmental barriers in rural areas (Maart *et al*. 2007). Societal perceptions, practices and ideologies form the basis of cultural beliefs, a known but less-explored barrier to accessibility of medical services in rural areas (MacLachlan 2006).

### Cultural beliefs

Cultural beliefs define who people are, how they interact with the world and how they behave in certain situations, and can be considered a combination of religious beliefs, socially accepted norms and traditions (Bailey, Erwin & Belin 2000; Omu & Reynolds 2012; Maart *et al*. 2007). Culture plays a central role in health related behaviours (Carroll *et al*. 2007; Omu & Reynolds 2012). The importance of cultural beliefs regarding health and health seeking behaviour has been well-documented (Bailey *et al*. 2000; Carroll *et al*. 2007; Legg & Penn 2013; Maart *et al*. 2007).

Different cultural groups have vastly different perceptions of the causes of disability and disease and these perceptions influence their health seeking behaviour (Bailey *et al*. 2000; Legg & Penn 2013; Pronyk *et al*. 2001). According to the South African Department of Health's Disability Survey, 3% of the population stated ‘bewitchment’ as the cause of their disability (DOH 2002). In a rural South African study the belief that ‘bewitchment’ caused tuberculosis resulted in a delay in seeking Western health care (Pronyk *et al*. 2001). Omu and Reynolds (2012) conducted a similar study into health seeking behaviours in Kuwait, and although persons with disabilities believed that their disability had a divine origin it did not stop them from utilising rehabilitation services. It is thus imperative to understand how a specific cultural group's beliefs influence their health seeking behaviour.

### Rehabilitation

Rehabilitation plays an essential role in minimising the impact of impairments on the activities of daily life and participation in their communities of persons with disabilities (World Health Organisation [WHO] 2011). Rehabilitation is also commonly used as an umbrella term for the therapy provided by different therapists and rehabilitation workers working together towards the common goal of improved functionality and quality of life for the person living with the disability. Physiotherapists have an important role to play in primary, secondary and tertiary prevention of disability in developing countries (Wickford & Duttine 2013). Rehabilitation is ideally provided by a multidisciplinary team, but most often physiotherapists or occupational therapists are the only rehabilitation workers servicing rural areas (Bateman 2012; WHO 2011). Human resources for health have been identified as a key priority for rural health care in South Africa (Versteeg, Du Toit & Couper 2013). Attracting and retaining staff to work in rural areas is a problem worldwide, and understanding the difficulties health care professionals face in these settings, is imperative to implementing retention strategies (Rural Health Advocacy Project [RHAP] & Partners 2013).

### Therapists’ perspective

Exploring the perspectives of the rehabilitation therapists aids in the understanding of the challenges they face as well as gaining insight into difficulties with rural rehabilitation. However, few studies explore the role of physiotherapists, occupational therapists and speech therapists working in rural areas and their views on factors that affect their services.

### Rationale

Health promoting programmes in developing countries are often not successful because of a lack of compatibility with culture specific beliefs (MacLachlan 2006).

Health care professionals’ lack of cultural awareness may lead to cultural imposition (Campinha-Bacote 2002). In order to provide an effective and culturally responsive health care service to the multi-cultural population of South Africa, health care workers need to be culturally aware and competent (Carroll *et al*. 2007; Campinha-Bacote 2002). According to Campinha-Bacote cultural awareness is a study of your own cultural biases and background in order to prevent imposing your own cultural beliefs on another cultural group. Cultural awareness is also considered the cornerstone to become culturally competent. According to Campinha-Bacote's (2002) model of cultural competence, becoming culturally competent consists of five constructs namely cultural awareness, knowledge, skill, encounters and desire of which cultural knowledge is a key factor. Cultural awareness relates to self-exploration and reflection on your own beliefs regarding culture. This process is an important step in trying to recognise your own biases in order to avoid imposing your own cultural beliefs on others. Cultural knowledge can be built by engaging with persons from different cultural backgrounds. The knowledge component that this article relates to is an understanding of a specific cultural group's worldview of their disability or disease, and how they make decisions regarding their own health. Cultural skill refers to the health care provider's ability to perform a physical assessment of a patient taking into consideration variations within different cultural groups. Cultural encounters and desire refer to the individual's initiative to experience difference cultures (Campinha-Bacote 2002). This article intends to enhance cultural awareness by the exploration of therapists’ perceptions about cultural beliefs.

The fact that cultural beliefs often lead to discrimination against persons with disabilities, has been covered in the literature. In a study on the abuse of disabled children in Ghana, the cultural belief that disabled children were cursed, led to such severe stigmatisation that children were often hidden away by their parents, or left at a river to die (Kassaha *et al*. 2012). However, more needs to be known about the perspectives of physiotherapists, occupational therapists and speech therapists on factors that affect their rehabilitation services in rural areas (Bateman 2012). Cultural beliefs can be considered as personal factors within the International Classification of Functioning, Disability and Health (ICF) framework that could potentially disable a person with an impairment. Identifying personal and contextual barriers that are associated with cultural beliefs will assist in minimising activity limitations and promote the integration of persons with disability into society (WHO 2001).

The aim of this study was to explore the experiences of rehabilitation therapists (physiotherapists, occupational therapists and speech therapists) working in a rural area in Kwazulu-Natal (KZN). The theme of cultural beliefs as a barrier to rehabilitation emerged so strongly in every focus group discussion, that it was explored in more depth with probing questions. This article primarily reports on the perceived effect of cultural beliefs on the utilisation of rehabilitation services in a rural community, potentially raising cultural awareness amongst therapists. Although the patients’ perspective could be considered a more accurate view of the beliefs that affect their utilisation of rehabilitation services, the view of experienced therapists working in a rural area is also an important consideration. The therapists’ views might be biased, but provide insight into their perceptions of cultural beliefs, and are important for improving rehabilitation services (Suddick & De Souza 2007). Raising cultural awareness amongst therapists working in rural areas could begin to address some of the many contextual factors inhibiting patients from accessing rehabilitation.

## Methodology

### Research question

Do cultural beliefs affect the utilisation of rehabilitation services in a rural community in South Africa?

### Aim

To explore the cultural beliefs that affect the utilisation of rehabilitation services in a rural community in South Africa from the therapists’ perspective.

## Design

An explorative qualitative design was utilised because very little information is available on the topic, and the problem is not well understood (Berg 2001). The primary method of data collection was focus group discussions (FGDs). A focus group uses a guided, interactional discussion as a means of formulating the details of complex experiences and the reasoning behind individuals’ actions, beliefs, perceptions and attitudes (Powell & Single 1996). Demographic information was also obtained and documented for each participant at the start of the focus group, but will not be published in order to respect the confidentiality of the participants.

### Setting

This study was conducted in a rural district in the KZN province of South Africa. The population in this district mainly represents the Zulu cultural group. Rurality is poorly defined in the South African context, but is generally classified according to the lack of infrastructure found in urban areas such as tarred roads, running water and electricity supply (Department of Provincial and Local Government [DPLG] 2000). Duncan, Sherry and Watson (2011:30) define rurality as the combination of multiple factors affecting the quality of life of people living in sparsely habituated settlements with limited access to public services. The Rural Doctors Association of South Africa (2006) considers an area ‘rural’ when more than 50% of the population lives further than five kilometres from a tarred road, and 25% of the population has to collect water from natural sources.

### Participants

The sampling frame for the study consisted of all rehabilitation therapists working at five district hospitals in a rural community in South Africa. All available therapists who agreed to participate at the time of the discussion were included. A total of 17 rehabilitation team members were conveniently selected to participate in the FGDs that were conducted at each of the five hospitals. The 17 participants included eight physiotherapists, seven occupational therapists, one dietician and one speech therapist.

### Data collection procedure

The head of the therapy department at each hospital was contacted telephonically, and appointments were made at a time that was convenient for most of the staff members, bearing disruption of their normal duties in mind. Data was collected by the researcher in person. The purpose and aim of the study was explained to all participants, and participating members signed an informed consent form agreeing to be audio-taped. Each participant completed the demographic survey. All five the FGDs were conducted at the therapy departments of the respective hospitals, and voice-recorded.

### Method of data collection

The focus group discussions were started with one grand question: ‘Can you please tell me more about your experiences as a rural therapist/rehabilitation team member?’. Participants freely shared any experience that they chose and the discussion flowed from the first participant's comments. The topic of how cultural beliefs affected the therapists’ experiences and especially the patients’ health seeking behaviour was raised by the therapists at each FGD, and probing questions were asked to explore this topic in more depth in subsequent FGDs. The fact that this topic was raised in every FGD without initial prompting from the researcher enhanced the relevance of it to this rural area, and the importance of cultural awareness to the therapists.

## Data analysis

The qualitative data was analysed using Creswell's (2009:185) eight step process of analysis. According to Creswell, following these steps from working with raw data to interpreting the meaning of themes assists in validating the accuracy of the information obtained from qualitative research studies. The interviews were transcribed verbatim, and checked for any mistakes or missed words against the audio recording. Checking of transcripts improves the trustworthiness of the findings (Gibbs 2007). All the interviews were conducted in English. The transcriptions were read and re-read several times by the researcher in order to gain an overall understanding of the data before commencing with the coding process. Making use of open and axial coding (Creswell 2009), transcripts were coded to identify common concepts within the participants’ responses. Codes were grouped into categories, and similar categories were analysed and emerging themes identified.

### Ethical considerations

Ethical clearance to conduct the study was obtained from the senate research committee at the University of the Western Cape. Permission was obtained from the relevant provincial Department of Health and the management of all the hospitals involved in the project. All participants gave informed consent in writing and agreed that their voices could be recorded. Participants were guaranteed that their identity would be kept confidential, and pseudonyms (P1–P17) were used in the transcription of the data instead of the participants own names. Only the researcher and the person who did the transcriptions had access to the voice-recordings. Participants were ensured that they could withdraw from the study at any time during the interviews without any consequences, and that they could inform the researcher if in hindsight they decided that what they had said could not be used for research purposes. No therapist made use of this opportunity or asked that anything that they shared should not be included in the study.

### Trustworthiness

A summary of each focus group discussion was sent back to the participants for review to establish that their comments were not misinterpreted by the researcher and to ensure dependability. The confirmability of the research was enhanced by asking an independent reviewer to analyse the raw data and compare the various categories and themes. An independent reviewer cross-checked the codes to determine inter-coder agreement and improve the trustworthiness of the findings (Creswell 2009). The specific findings of this study do not have high transferability, because the cultural beliefs mentioned in the study might only be representative of the specific cultural group. The general influence of cultural beliefs on utilisation of rehabilitation services might however be applicable to other cultural groups in rural regions of South Africa.

## Findings

### Participants

The mean age of the participants was 27 years at the time of data collection. The racial distribution of participants was almost equal. Nine Caucasian and eight African staff members participated in the discussions. Of the 17 participating therapists, 10 were female, and 7 were male. The mean years of experience working in a rural area was five years for permanent staff members, and three and a half years when taking the community service therapists (contract staff) into consideration.

According to therapists working in this area, cultural beliefs play a major role in the utilisation of rural health services. In this specific Zulu community different beliefs affecting rehabilitation services were identified. These beliefs were grouped into two themes: cultural beliefs preventing patients from accessing rehabilitation services, and cultural beliefs affecting the rehabilitation process of the patient when utilising the service.

### Cultural beliefs preventing the utilisation of rehabilitation services

Several cultural beliefs seemed to prevent patients from utilising rehabilitation services. These factors were categorised into beliefs about the cause of a disease, stigma and community perception of a person's worth.

### Beliefs regarding cause of disease

The therapists reported that patients believed that their pain and disease is of a spiritual nature and that Western medicine cannot cure them in the spiritual realm. This belief often leads to the refusal of hospital treatment, with patients opting to consult a ‘spiritual’ or traditional healer. One therapist reported patients saying:

- ‘I had a dream last night that somebody stood on me in my dream and now it's a curse that's been put on me [*therapist quoting a patient's description of how his pain started*] (P6)’.
- ‘…[*Y*]ou can't separate traditional and cultural factors from your treatment but you can do as much as you can in the hospital and if the family decides to go and consult with a traditional healer you can only advocate this much…you can't judge it either, you can't say you are doing the wrong thing and you are going to kill this person if you do this’ (P6).

### Stigma

Therapists highlighted that patients often do not attend therapy because it is too difficult for them to get to the hospital or clinic as a result of the stigma attached to being disabled. Taxis and cars will not stop to provide transport for persons with disabilities because they believe that the person might be cursed; so if people with disabilities do not own a car or is not able to drive themselves, they cannot attend therapy. According to Participant 4:

‘Some people discriminate against moms with disabled children because they are “strange” and they don't like having them in their cars … it's not always just money, its people's attitudes towards disabled people’.

### Community perception of worth

Persons with disabilities were perceived to be less valuable in their communities or household if they were dependent on carers and could not continue contributing towards the household. This was more evident when the patient did not receive a disability grant. Subsequently therapists reported that their condition often deteriorated at home:

‘…[*Y*]ou find, at home the people who are supposed to be looking after the patients, you know, usually lose that kind of care for the patient, because now the patient has to depend on them foreverything, so you find that most of those patients, their condition usually get worse’. (P10)

### Cultural beliefs affecting the utilisation of rehabilitation services

In some cases patients did commence rehabilitation, but cultural beliefs played an important role in the patients’ conviction regarding the efficacy, continuity and quality of rehabilitation, from the therapists’ perspective.

### Lack of conviction about the efficacy of rehabilitation

Patients did not belief that rehabilitation would be effective in decreasing their disability, because they do not understand the cause of their problem, or they believe that it has a mystical origin:

- ‘I had specifically a girl that has a psychological gait pattern, she was telling me that people don't want her to walk, and that people have cursed her … So we've sent her to the psychologist to see what it was, he told us that she has the ‘*ukuthwasa*’ or the calling to become a sangoma, and if you deny that calling, then it will manifest physically in your body as a disability. You can do whatever you want for her, but if she believes that this is ‘*ukuthwasa*’ and unless she goes that route it will not be sorted out (P11).
- To go now [*and advise the patient*] you need to go do these exercises after you [*the patient*] think you've got this pain because you had this dream or you've been bewitched or something is a very, very challenging thing’ (P6).

### Continuity of rehabilitation

Often patients come for rehabilitation but their cultural roles prevent them from complying with therapy or rehabilitation; for example, only women traditionally fetch and carry water on their heads. If a female injured her neck or suffers from arthritis, she is culturally not allowed to modify her behaviour in order to rehabilitate her injury. ‘Women are expected to “*twala*” [carry] everything so if they are sick they understand, but still continue with the work’ (P7).

Patients in rural areas also seemed to have a cultural misconception that therapists from their own culture were less qualified or less capable of providing a good service than therapists from other cultures. They would sometimes stop rehabilitation if they realised that the therapist was someone they knew from the area, or if they were from their own race:

- ‘…[*B*]ecause we are black, people they undermine us. If you are from the local area … they say, oh, you know me …’ (P8).
- ‘…[*S*]o that is the kind of perception that they'll have that …hey, you don't write on my file if you are, you know, my own race!’ (P10).
- ‘[*I*]t's just the kind of perception that they have, you know, the only educated person, you know is the white person, especially with the old age group [*older person*]’ (P10).

### Quality of rehabilitation

Most therapists felt that the quality of the rehabilitation services that they provided was compromised by cultural beliefs. This was true for therapists from the same and different cultures. Therapists from a different culture who did not speak the local language rely on translators to assist with the diagnosis and treatment of the problem. Therapists felt that the local translators modified what they said to the patients because they did not believe or understand what the therapist was trying to explain to the patient. ‘The way that the translators will translate … it's like you say this long like sentence and they [*the translator*] just say like two words … they don't understand’ (P1).

Cultural beliefs also made the provision of certain services impossible. Therapists felt that mental health problems were especially difficult to negotiate:

‘As an OT [*occupational therapist*] for mental health issues … I don't even go there because there are so many cultural beliefs involved … there's so many, to do that through a translator as well, I find it very difficult’ (P2).

Therapists became discouraged and felt that lack of conviction about the efficacy of therapy and lack of belief that therapy can improve the patient's quality of life negatively affected the quality of services they provided. 'Their [*the patients*’] attitudes [*towards the service*] affect the services we are giving … they don't see the meaning of what we are doing’ (P8).

## Discussion

The finding relating to the cultural belief that disability has a spiritual or mystic origin that was evident in this study corresponds with the findings of Madden *et al*. (2013), the DOH (2002), Carroll *et al*. (2007) and Bailey *et al*. (2000). All these studies noted that in many cultures people still believe that their disability or disease manifests itself as a result of wrongdoing against their ancestors, and are spiritual in origin. Legg and Penn (2013) also reported that patient explanations of the causes of aphasia after a stroke were strongly influenced by cultural beliefs. They report that patients with aphasia believed that misfortune or other spiritual causes resulted in them having a stroke and subsequently aphasia. This perception of the cause of the condition negatively affected this population's health seeking behaviour. In a study conducted by Kassaha *et al*. (2012:695) in Ghana regarding abuse of disabled children, these children were often killed based solely on the cultural belief that they were ‘supernatural’ or ‘cursed beings’. These studies support the perception of therapists in this study that patients’ cultural beliefs regarding their disability play an important role in the utilisation of, and belief in, the efficacy of rehabilitation.

The establishment in this study that beliefs regarding aetiology of diseases affect health seeking behaviours correspond with the findings of Pronyk *et al*. (2001). In his South African study on the health seeking behaviour of patients with tuberculosis, Pronyk reported that the patients’ cultural beliefs regarding the aetiology of tuberculosis was a strong barrier to the utilisation of health care services.

Cultural beliefs regarding the causes of disability do not only affect the health seeking behaviour of patients living in rural areas, but also their conviction about the effectiveness of therapy services. Therapists reported that if patients believed that their disability was caused by an ancestral curse, the patients would not comply with doing exercises because they would not be able to rationalise how it could remove the curse in order to heal them. This finding is resonated by Madden *et al*. (2013) who reported that physiotherapists had very little success in treating patients with lower back pain in a very similar rural setting. One of the reasons given for this finding in this study, was because of the patients’ cultural beliefs regarding the cause of their back pain. It was noted that patients rarely adopted the suggested exercises or treatments since they did not believe in the efficacy of it, in light of their beliefs regarding the cause of their pain.

Therapists expressed the view that cultural expectations affected compliance with therapy. In order to relieve the patient's pain, the therapist has to ask the patient to make certain lifestyle changes or modify their behaviour, for example, to stop carrying heavy buckets of water on their heads. The cultural expectation in the Zulu culture is that it is the female's responsibility to fetch and carry water (Madden *et al*. 2013). Madden also described similar findings in relation to females with back pain carrying heavy buckets of water on their heads. The role of the female in the traditional Zulu culture is to serve her husband, and care and provide for her family. Therapists found that patients simply could not comply with recommendations to stop carrying heavy 25 litres buckets on their heads due to cultural expectations (Madden *et al*. 2013). Even though cultural roles and beliefs could play a role in preventing lifestyle changes, socio-economic factors and structural poverty could also affect these decisions (Bohrat & Kanbur 2006). Lack of access to running water might force a female to continue fetching water especially if the males are working in cities as migrant workers as it is often the case in rural areas (Coovadia *et al*. 2009).

Madden *et al*. (2013) reported that physiotherapists in South Africa were generally ill-trained and unprepared for the cultural and contextual factors that influence rehabilitation in rural areas. Culture specific knowledge regarding the aetiology of disease in rural communities is vital in promoting rehabilitative services in these areas. The cultural beliefs of this specific community also impacted negatively on the perceived quality of rehabilitation provided by the therapists. In this rural district in South Africa there is still a tremendous need for health education regarding the cause of disability where very few persons with disabilities are currently seeking rehabilitation. Therapists do recognise that cultural beliefs regarding the aetiology of their disability are only one of many barriers to accessing rehabilitation services in this setting. The list of environmental barriers such as lack of infrastructure, the poor public transport system, high unemployment rates and poverty are all factors limiting accessibility (Madden *et al*. 2013; Maart *et al*. 2007).

All therapists were aware of these factors and indicated that a community based rehabilitation (CBR) approach would be far more beneficial in meeting the needs of the community (WHO 2010). They did, however, note that staff shortages and lack of vehicles for therapists to do home and clinic visits were amongst the main barriers to implementing a more effective CBR programme. Currently no new community health workers are being employed or trained in order to implement CBR in this rural district. Only community caregivers were employed by the DOH, and they were not trained or allowed to do CBR. In one of the rural hospitals eight therapists (four permanent and four community service therapists) are employed to service approximately 100 000 people over a surface area of 3000 square kilometres (Kwa-Zulu Natal Department of Health [KZN DOH] 2001b). This amounts to a therapist to patient ratio of 1 to 12 500, with each therapist being responsible for approximately 375 square kilometres of rural surface area. At this specific hospital therapists were aware of one community health worker that was still employed by the hospital to cover all 3000 square kilometres – and this person was also blind. Because of the large distances that have to be covered in order to provide an effective CBR service, more trained staff and community health workers as well as vehicles that can accommodate rural terrain will be beneficial.

Stigmatisation of persons with physical disabilities is well-documented (Bagenstos 2000; McMaugh 2011; Tyrrell *et al*. 2010; Wang & Dovidio 2011). According to Bagenstos, society has historically discriminated against persons with disabilities based on their ‘abnormal’ appearance. In this study, therapists identified that stigmatisation of persons with disabilities made it very difficult for these patients to obtain transport to attend therapy. The prejudice against persons with disabilities in this study also seemed to be largely related to the fact that they looked different and as a result the cultural belief that a person was ‘bewitched’.

Accessibility and lack of transport is a major barrier to the utilisation of medical services in rural communities (Gallagher *et al*. 2011; Goins *et al*. 2005; Gordon 2009; Maart *et al*. 2007) and in this study therapists specifically mentioned that people would not allow persons with disabilities to make use of public transport due to the stigma. In some of the communities, persons with disabilities would be allowed onto the taxi, but would have to pay double if they had a wheelchair or even an assistive device. Therapists mentioned that the ability to pay the extra fee sometimes stopped patients from returning for therapy; especially mothers of children with cerebral palsy would simply not even be allowed into an empty taxi. According to the therapists, persons with physical disabilities were more stigmatised than those with visual or mental impairments. This is, however, an area that could be explored in more depth. Persons with disabilities living in rural areas are doubly disadvantaged with regards to their ability to access rehabilitation services. The geographical as well as the attitudinal environment (WHO 2001) were barriers to them accessing rehabilitation services. More importantly, re-engineering of primary health care (PHC) should ideally include a policy shift by DOH towards structural and intersectoral support for community based rehabilitation (WHO 2010). Such support would release therapists from hospitals to work at the coalface in people's lived environments. It would also include the development of a cadre of rehabilitation community workers to deliver home based services under the direction of rehabilitation therapists.

Therapists also reported that patients usually deteriorated at home once they were discharged from the hospital. They attributed the patient's deterioration to the cultural belief that a person with a disability could not contribute to the household and was not worthy of care and limited financial resources. This finding directly contradicts recent disability literature which states that persons with disabilities are valued as a result of their potential to qualify for disability grants of approximately R1200 per month (Leclerec-Madlala 2006; Penn 2014). The fact that the therapists discussed this issue could either indicate that not all rural families are aware of disability grants, or possibly cannot access it due to problems with the system or lack of personal identification documents (ID) (Penn 2014; *Social Assistance Act* 2004). According to the *Social Assistance Act* (2004) an ID is a requirement for applying for a disability grant. Therapists did mention that they would encourage persons with disabilities to apply for a disability grant, but that obtaining an ID is a challenge when people have to travel to the nearest Department of Home Affairs to apply for it. Poverty and a poor public transport system are noted in the literature as some of the main reasons why patients do not return for follow-up visits (Gallagher *et al*. 2011; Maart *et al*. 2007). As discussed earlier, this problem could potentially be addressed if basic resources such as transport which could accommodate rural terrain was available for therapists to provide a service that is more aligned with a CBR approach.

An interesting observation that could be unique to the South African context was that patients were resistant to being treated by therapists from their own culture and race. This finding is contrary to the Rural Health Strategy for South Africa (DOH 2006) which advocates the training of local people to strengthen the health care workforce in rural areas. The therapists in this study reported that patients believed that only white people could be educated enough to be doctors and therapists and that their ‘own people’ were not seen as competent. This belief could also be unique to the area since most of the rural hospitals in this district originated as ‘missionary hospitals’ that were predominantly staffed by European volunteers (KZN DOH 2001a; 2001b).

Therapists also felt that the quality of their rehabilitation services was negatively impacted on by the cultural beliefs of their patients. They felt that since some of them had to make use of translators who would change what they said to fit the cultural context, they could not educate or counsel patients sufficiently. The value of using formally trained interpreters in cross cultural encounters is reiterated by Campinha-Bacote (2002). This finding is resonated by Carroll *et al*. (2007:362) who notes that it is often difficult to provide health care services making use of translators as the ‘… translation may not fully represent cross-cultural differences in conceptualizations of health’.

## Recommendations

Therapists intending to follow a career in rural health care – or even ‘community service therapists’ – should be aware and sensitive to the cultural beliefs that could potentially have an impact on their services. Currently rehabilitation therapists working in this area try and do ‘roadshows’. These events serve to educate the community about the causes of disability and to raise awareness about the importance of rehabilitation. If therapists are more aware of how cultural beliefs could affect the utilisation of their services, they could potentially assist in changing cultural perceptions about health. Unfortunately as a result of staff shortages in rural areas this does not happen often. It is vital to advocate for the attraction and retention of more rehabilitation therapists to work in rural areas in order to facilitate a more effective CBR approach. Decentralisation of rehabilitation services will improve the utilisation of services by removing some of the structural poverties which undoubtedly affect access to rehabilitation.

### Limitations of the study

The views explored in this study are only representative of the rehabilitation therapists working in rural hospitals and not necessarily the only reason for poor utilisation of rehabilitation services, but are in their perception a major contributing factor in this specific area.

## Conclusion

In this study from the therapists’ perspective, the cultural beliefs regarding the aetiology of disease and disability impacted negatively on the utilisation of rehabilitation services. This finding provides valuable insight into the perceptions of the therapists working in this rural community. Their perceptions on how cultural beliefs affect the utilisation of their services can also assist to inform education and health promotion programmes specifically in a rural South African context.

It is the responsibility of all health care providers to ensure that they become culturally aware, knowledgeable and competent in order to provide the best possible services that meet the needs of the intended community. It is also a call to institutions of higher education to better prepare undergraduate health care professionals for working in the rural context as well as the national DOH to consider providing the necessary human and structural resources which could assist therapists to follow a CBR approach and truly provide ‘health for all’.
